# Supplementary material for: Thiobarbiturate-Derived Compound MHY1025 Alleviates Renal Fibrosis by Modulating Oxidative Stress, Epithelial Inflammation, and Fibroblast Activation
Source: Antioxidants (Basel). 2023 Oct 31;12(11):1947. doi: 10.3390/antiox12111947 (PMC10669799; doi:10.3390/antiox12111947)
Supplement: Supplementary file 1 [file antioxidants-12-01947-s001.zip › antioxidants-2648626-supplementary.pdf]

Supplementary Table 1. Information of Antibodies used in Western blotting

Antibody	Company	Catalog number
α-SMA	Santa Cruz	sc 32251
NF-κB (p65)	Santa Cruz	sc 514451
Phospho-NF-κB (p-p65)	Santa Cruz	sc 13548
CD3	Santa Cruz	sc 20047
CD68	Santa Cruz	sc 20060
COL1A2	Santa Cruz	sc 393573
α-Tubulin	Santa Cruz	sc 5286
Gapdh	Santa Cruz	sc 365062
Lamin A/C	Santa Cruz	sc 376248

Supplementary Table 2. Primer sequences for qPCR

Mouse

Gene	*Forward (5'-3')*	*Reverse (3'-5')*
Col1a2	*CAGCTCCAGGAAGACCTCGA*	*GTAACAAGGGTGAGCCTGGC*
Col3a1	*GGTGGCTGCATCCCAATTCA*	*GGCAGGGACAACTGATGGTG*
Vim	*CAAGCCTGACCTCACTGCTG*	*CACCTGTCTCCGGTACTCGT*
Tgfb1	*CCTCACCTCCATGTACCAGAA*	*TGGAAATGACCTTGTCAATGAG*
Fn	*CAACAACCGGAATTACACCG*	*GTCTCGGAGCTGGGAGTAGG*
Acta2	*TTGCTGACAGGATGCAGAAG*	*TGATCCACATCTGCTGGAAG*
Ccl2	*CCAGCAAGATGATCCCAATG*	*CTTCTTGGGGTCAGCACAGA*
Ccl5	*CCCTCACCATCATCCTCACT*	*CCTTCGAGTGACAAACACGA*
Ccl7	*TGAAAACCCCAACTCCAAAG*	*CATTCCTTAGGCGTGACCAT*
Cxcl1	*AATGCATCCACATGCTGCTA*	*ATAGCCTCCTCGACCCACTT*
Emr1	*TCTGGGGAGCTTACGATGGA*	*GAATCCCGCAATGATGGCAC*
Cd68	*GGGGCTCTTGGGAACTACAC*	*GTACCGTCACAACCTCCCTG*
Gapdh	*AAGGTCATCCCAGAGCTGAA*	*CTGCTTCACCACCTTCTTGA*

Rat

Gene	*Forward (5'-3')*	*Reverse (3'-5')*
Ccl2	*GCCAACTCTCACTG AGCCA*	*GCATCTGGCTGAGACAGCAC*
Cxcl1	*GCTGGGATTCACCTCAAGAA*	*TGGGGACACCTTTTAGCATC*

Il8	*GAAGATAGATTGCACCGA*	*CATAGCCTCTCACACATTTC*
Tgfb	*ATACGCCTGAGTGGCTGTCT*	*TGGGACTGATCCCATTGATT*
Acta2	*ACTGGGACGACATGGAAAAG*	*CATCTCCAGAGTCCAGCACA*
Col1a2	*TCAAGGTTTCCAAGGACCTG*	*TTTCCAGGGTGACCATCTTC*
Col3a1	*AGCTGGACCAAAAGGTGATG*	*TCCAGTTAGCCCTGCAATTC*
Fn	*GCTGTGCCTTCATTGTTCAG*	*CCATTCATGAGAAGCAGCAG*
Vim	*TGCACGATGAAGAGATCCAG*	*TGGCAGCCACACTTTCATAC*
18s	*AGTCGGCATCGTTTATGGTC*	*CGCGGTTCTATTTTGTTGGT*
Gapdh	*ACAGCTGCTGCTTTCACCGT*	*TCAACCCACTTCTGATGGGCT*
